# Balloon kyphoplasty versus percutaneous vertebroplasty for osteoporotic vertebral compression fracture: a meta-analysis and systematic review

**DOI:** 10.1186/s13018-018-0952-5

**Published:** 2018-10-22

**Authors:** Bo Wang, Chang-Ping Zhao, Lian-Xin Song, Lian Zhu

**Affiliations:** Department of Orthopedic Trauma Centre, 3rd Hospital of Hebei Medical University, No. 139 ZiQiang Road, Qiaoxi District, Shijiazhuang, 050051 China

**Keywords:** Meta-analysis, Balloon kyphoplasty, Percutaneous vertebroplasty, Osteoporotic vertebral compression fracture

## Abstract

**Background:**

This meta-analysis was aimed to explore the overall safety and efficacy of balloon kyphoplasty versus percutaneous vertebroplasty for osteoporotic vertebral compression fracture (OVCF) based on qualified studies.

**Methods:**

By searching multiple databases and sources, including PubMed, Cochrane, and Embase by the index words updated to January 2018, qualified studies were identified and relevant literature sources were also searched. The qualified studies included randomized controlled trials, prospective or retrospective comparative studies, and cohort studies. The meta-analysis was performed including mean difference (MD) or relative risk (RR) and 95% confidence interval (95% CI) to analyze the main outcomes.

**Results:**

A total of 16 studies were included in the meta-analysis to explore the safety and efficacy of kyphoplasty versus vertebroplasty for the treatment of OVCF. The results indicated that kyphoplasty significantly decreased the kyphotic wedge angle (SMD, 0.98; 95% CI 0.40–1.57), increased the postoperative vertebral body height (SMD, − 1.27; 95% CI − 1.86 to − 0.67), and decreased the risk of cement leakage (RR, 0.62; 95% CI 0.47–0.80) in comparison with vertebroplasty. However, there was no statistical difference in visual analog scale (VAS) scores (WMD, 0.04; 95% CI − 0.28–0.36) and Oswestry Disability Index (ODI) scores (WMD, − 1.30; 95% CI − 3.34–0.74) between the two groups.

**Conclusions:**

Kyphoplasty contributes especially to decreasing the mean difference of kyphotic wedge angle and risk of cement leakage and increasing the vertebral body height when compared with vertebroplasty. But radiographic differences did not significantly influence the clinical results (no significant difference was observed in VAS scores and ODI scores between the two groups); thus, kyphoplasty and vertebroplasty are equally effective in the clinical outcomes of OVCF. In addition, more high-quality multi-center RCTs with a larger sample size and longer follow-up are warranted to confirm the current findings.

## Background

Osteoporotic vertebral compression fracture (OVCF), one of the most common healthcare issues worldwide, commonly occurs after ankle, wrist, or hip fractures. Its incidence and severity have been steadily increasing over the last decades among elderly patients. In the USA, approximately 750,000 adults suffer from OVCFs each year [1], including 8% of women older than 50 years of age and 27% of men and women older than 65 years of age [2]. However, only about one third of patients with fractures are symptomatic, with compromised quality of life. OVCF occurs due to insufficient anterior vertebral height and causes spinal deformities, reduced pulmonary function, restriction of the abdominal and thoracic contents, impaired mobility, and depression [3–5]. Moreover, it prolongs hospitalization, affects quality of life, increases morbidity, and inflicts a heavy burden on the society.

Different approaches are available for the treatment of OVCFs, including standard medical and surgical therapy. The standard medical therapy contains bed rest, analgesia, bracing, external fixation, rehabilitation, and a combination of these treatments [6]. However, there are several limitations in the standard therapy: long-term bed rest can lead to subsequent demineralization and OVCF recurrence; anti-inflammatory drugs and certain types of analgesics cause intolerable side effects for older patients; and medical management does not reverse kyphotic deformity. Surgical treatment involves surgical stabilization via dorsal instrumentation, which is available for patients with OVCFs who are refractory to medical therapy [7]. Due to the poor quality of the osteoporotic bone, classical open surgery with metal implants often fails and leads to persistent back pain, neurological symptoms, and limited functions [8, 9]. Vertebroplasty was introduced by Galibert and Deramend in 1984 in France for treating hemangiomas at the C2 vertebra [10]. Balloon kyphoplasty was first performed in 1998. It is a minimally invasive surgical technique that corrects kyphosis secondary to collapsed vertebral bodies using a balloon (an inflation bone tamp) [9].

Therefore, in this meta-analysis, we assessed the existing evidence on the safety and effect of balloon kyphoplasty versus vertebroplasty in the treatment of OVCFs based on qualified trials.

## Methods

### Search strategy

The Cochrane Library, PubMed, and Embase databases were searched updated to January 2018 for all the qualified studies in order to analyze the effect of balloon kyphoplasty versus vertebroplasty in the treatment of OVCF. Literature was also identified by tracking reference lists from papers and Internet searches. Two investigators independently extracted data, and a third investigator was involved when a disagreement occurred.

### Study selection

To be included in the meta-analysis, studies should meet the following criteria: (1) comparative studies: randomized controlled trails, prospective or retrospective case-control study, or cohort study; (2) the included patients had OVCF; (3) the test group were treated with balloon kyphoplasty the control group were treated with vertebroplasty; (4) the clinical outcomes included the visual analog scale (VAS) scores, Oswestry Disability Index (ODI), kyphotic wedge angle, vertebral body height restoration, and incidence of cement leakage; and (5) the publications were available in English and Chinese.

The following studies were excluded from the review: (1) repeat published articles or articles having the same content and result; (2) case report, theoretical research, conference report, systematic review, meta-analysis, expert comment, and economic analysis; (3) the outcomes were not relevant.

### Data extraction

Two reviewers determined study eligibility independently. A third investigator was involved to reach an agreement. The analyzed data were extracted from all the included studies and consisted of two parts: basic information and main outcomes. The first part was about the basic information: the authors’ name, the publication year, study design, country, sample size, age, and percentage of male. The second part was the clinical outcomes: the VAS scores, ODI, kyphotic wedge angle, vertebral body height restoration, and incidence of cement leakage. The studies were performed by two reviewers independently. Any arising difference was resolved by discussion.

### Statistical analysis

All statistical analyses were performed in the STATA 10.0 (TX, USA). Chi-squared and *I*^2^ tests were used to assess heterogeneity of clinical trial results and determine the analysis model (fixed-effects model or random-effects model). When the chi-squared test *P* value was ≤ 0.05 and *I*^2^ tests value was > 50%, it was defined as high heterogeneity and assessed by the random-effects model. When the chi-squared test *P* value was > 0.05 and *I*^2^ tests value was ≤ 50%, it was defined as an acceptable heterogeneity data and assessed by the fixed-effects model. Continuous variables were expressed as mean ± standard deviation and analyzed by mean difference (MD). Categorical data were presented as percentages and analyzed by relative risk (RR) or odds ratio (OR). VAS, ODI, the kyphotic wedge angle, and the vertebral body height were analyzed by MD and 95% confidence interval (CI). The incidence of cement leakage was analyzed by RR and 95% CI.

## Results

### Characteristics of the included studies

**Fig. 1:**
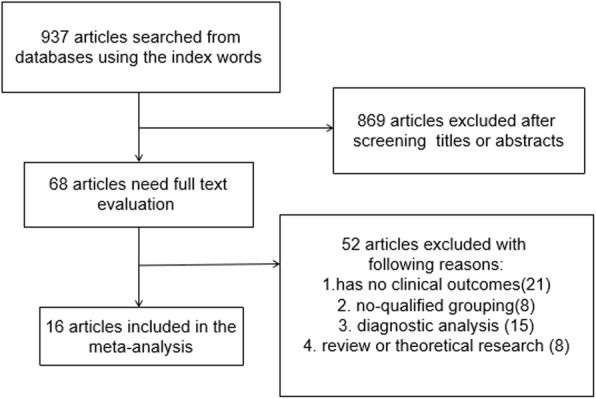
Flow diagram of the literature search and selection process

By searching multiple databases and sources, we identified 937 articles by the index words. After screening titles and abstracts, 869 articles were excluded, leaving 68 articles for further evaluation. During full-text screening, 52 articles were excluded due to the following criteria: for having no clinical outcomes (*n* = 21), no qualified outcomes (*n* = 8), diagnostic analysis (*n* = 15), and theoretical research or review (*n* = 8). Finally, 16 studies [11–26] were included in the meta-analysis with 647 subjects in the kyphoplasty group and 758 subjects in the vertebroplasty group. The selection process is presented in Fig. 1.

The main characteristics of the included studies are summarized in Table 1, with one prospective randomized comparative study, four prospective comparative studies, five prospective cohort studies, two retrospective comparative studies, and four retrospective cohort studies. The patients were from Israel, Australia, Japan, Canada, Italy, Slovenia, the USA, Spain, Germany, China, and Korea. The age of patients in the kyphoplasty group and the vertebroplasty group was all more than 60 years. Other information included the number of patients and gender.

**Table 1:** The basic characteristics description of included studies

Study	Study design	Country	No. of patients		Age		Gender	
			KP	VP	KP	VP	KP	VP
Yoram Folman 2011 [11]	Prospective cohort	Israel	31	14	70.74	75.57	9 M	5 M
Grohs JG 2005 [12]	Prospective comparative study	Australia	28	23	70		7 M	5 M
A. Hiwatashi 2009 [13]	Retrospective cohort	Japan	40	66	75	77	11 M	21 M
Krishna Kumar 2010 [14]	Prospective cohort	Canada	24	48	73	78	7 M	9 M
J. T. Liu 2009 [31]	Prospective randomized comparative study	Taiwan	50	50	72.3	74.3	11 M	12 M
Alessio Lovi 2009 [16]	Prospective cohort	Italy	36	118	67.6		98 M	
I. Movrin 2010 [17]	Prospective cohort	Slovenia	46	27	67.8	72.9	10 M	5 M
M. Rollinghoff 2009 [18]	Prospective comparative study	USA	53	52	68.9		20 M	
Fernando Ruiz Santiago 2010 [19]	Prospective cohort	Spain	30	30	65.9	73	9 M	5 M
Markus Dietmar Schofer 2009 [20]	Prospective comparative study	Germany	30	30	72.5	73.8	8 M	6 M
Denglu Yan 2011 [21]	Retrospective cohort	China	98	94	76.9	77.2	41 M	39 M
Zhou Jianlin 2008 [22]	Retrospective cohort	China	42	56	64	62	17 M	21 M
Hu Chunhua 2016 [23]	Retrospective cohort	China	30	30	67.44	68.73	18 M	18 M
Du Junhua 2014 [24]	Prospective comparative study	China	44	42	75.6	72.1	8 M	9 M
Wu Yao 2014 [25]	Retrospective comparative study	China	20	20	65.12	66.37	9 M	12 M
Kyung-Hyun Kim 2012 [26]	Retrospective comparative study	Korea	45	58	72.5	74.6	10 M	13 M

### VAS

**Fig. 2:**
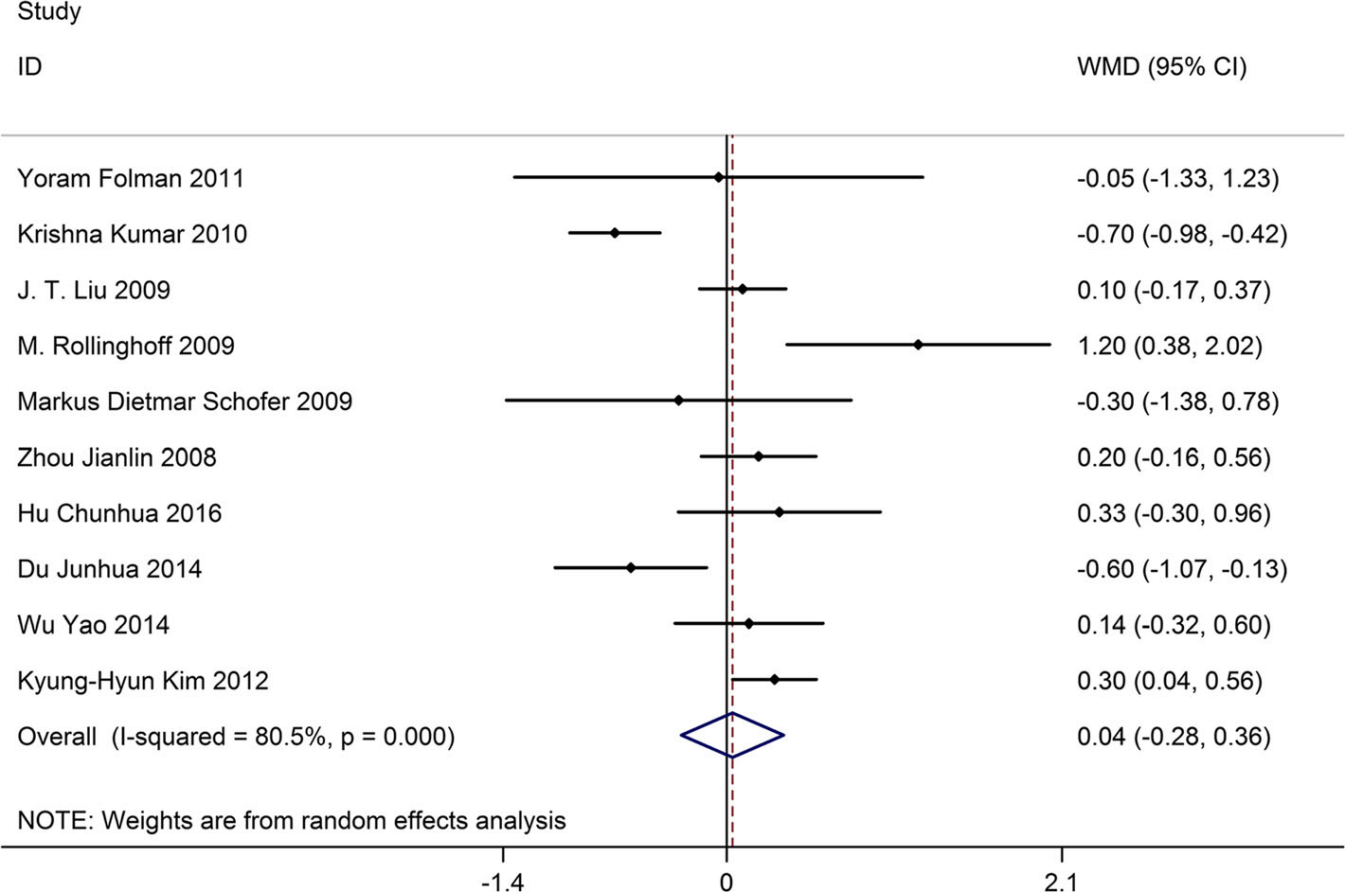
Forest plot showing the mean difference of VAS scores between kyphoplasty and vertebroplasty

Ten studies on 769 patients provided preoperative and postoperative VAS scores. Based on the chi-squared test *P* value (*P* = 0.000) and *I*^2^ test value (*I*^2^ = 80.5%), we chose the random-effects model to analyze the MD of VAS scores between kyphoplasty and vertebroplasty. The pooled results showed no significant difference in changes in VAS scores between kyphoplasty and vertebroplasty (WMD, 0.04; 95% CI − 0.28–0.36, Fig. 2).

### ODI

**Fig. 3:**
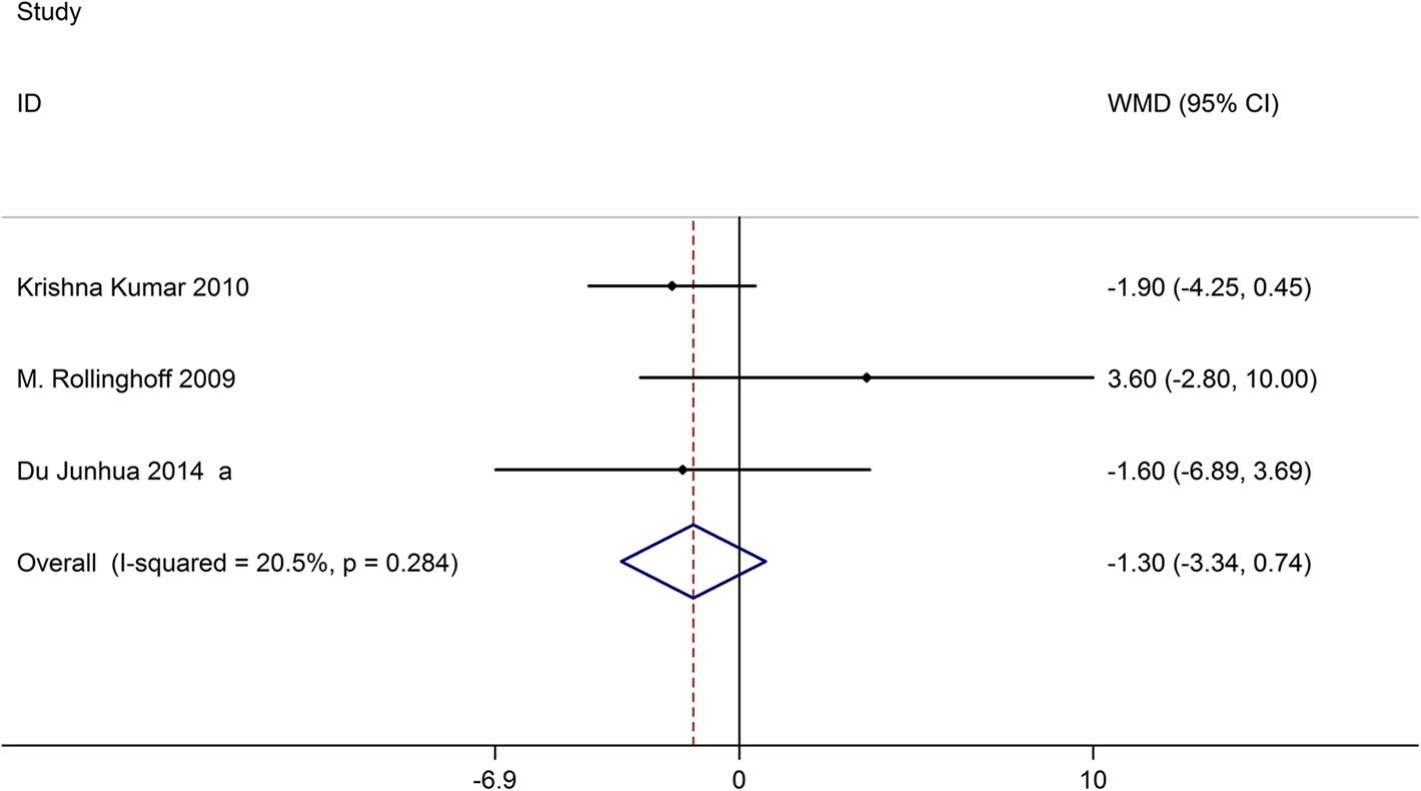
Forest plot showing the mean difference of ODI scores between kyphoplasty and vertebroplasty

Three studies on 263 patients had preoperative and postoperative ODI scores. Based on the chi-squared test *P* value (*P* = 0.284) and *I*^2^ test value (*I*^2^ = 20.5%), we chose the fixed-effects model to analyze the MD of ODI scores between kyphoplasty and vertebroplasty. The pooled results showed no significant difference in changes in ODI scores between kyphoplasty and vertebroplasty (WMD, − 1.30; 95% CI − 3.34–0.74, Fig. 3).

### Kyphotic wedge angle

**Fig. 4:**
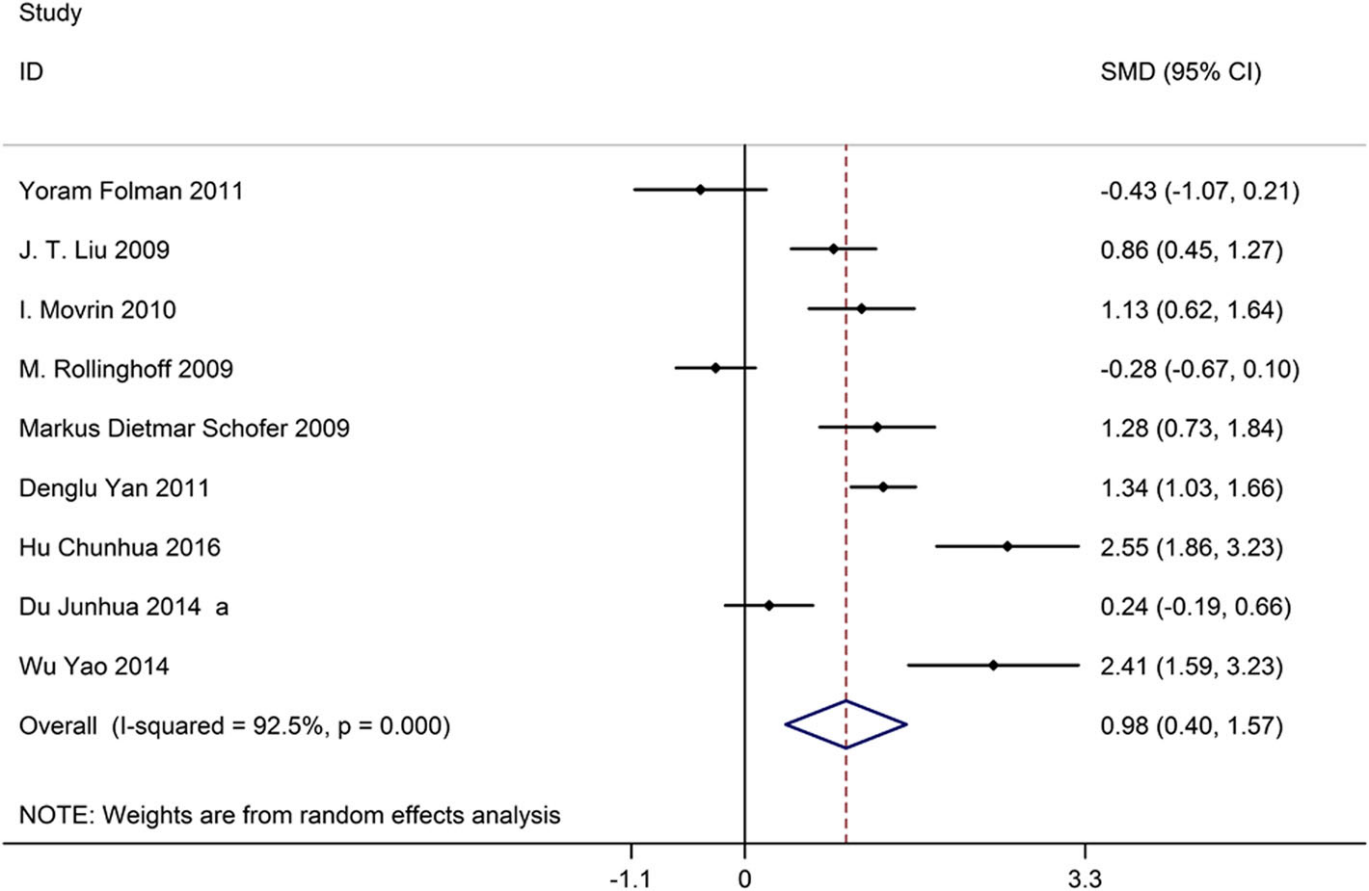
Forest plot showing the mean difference of kyphotic wedge angle between kyphoplasty and vertebroplasty

Nine studies on 761 patients had preoperative and postoperative kyphotic wedge angles. Based on the chi-squared test *P* value (*P* = 0.000) and *I*^2^ test value (*I*^2^ = 92.5%), we chose the random-effects model to analyze the MD of the kyphotic wedge angle. The pooled results showed that compared with vertebroplasty, kyphoplasty significantly decreased the kyphotic wedge angle (SMD, 0.98; 95% CI 0.40–1.57, Fig. 4).

### Vertebral body height

**Fig. 5:**
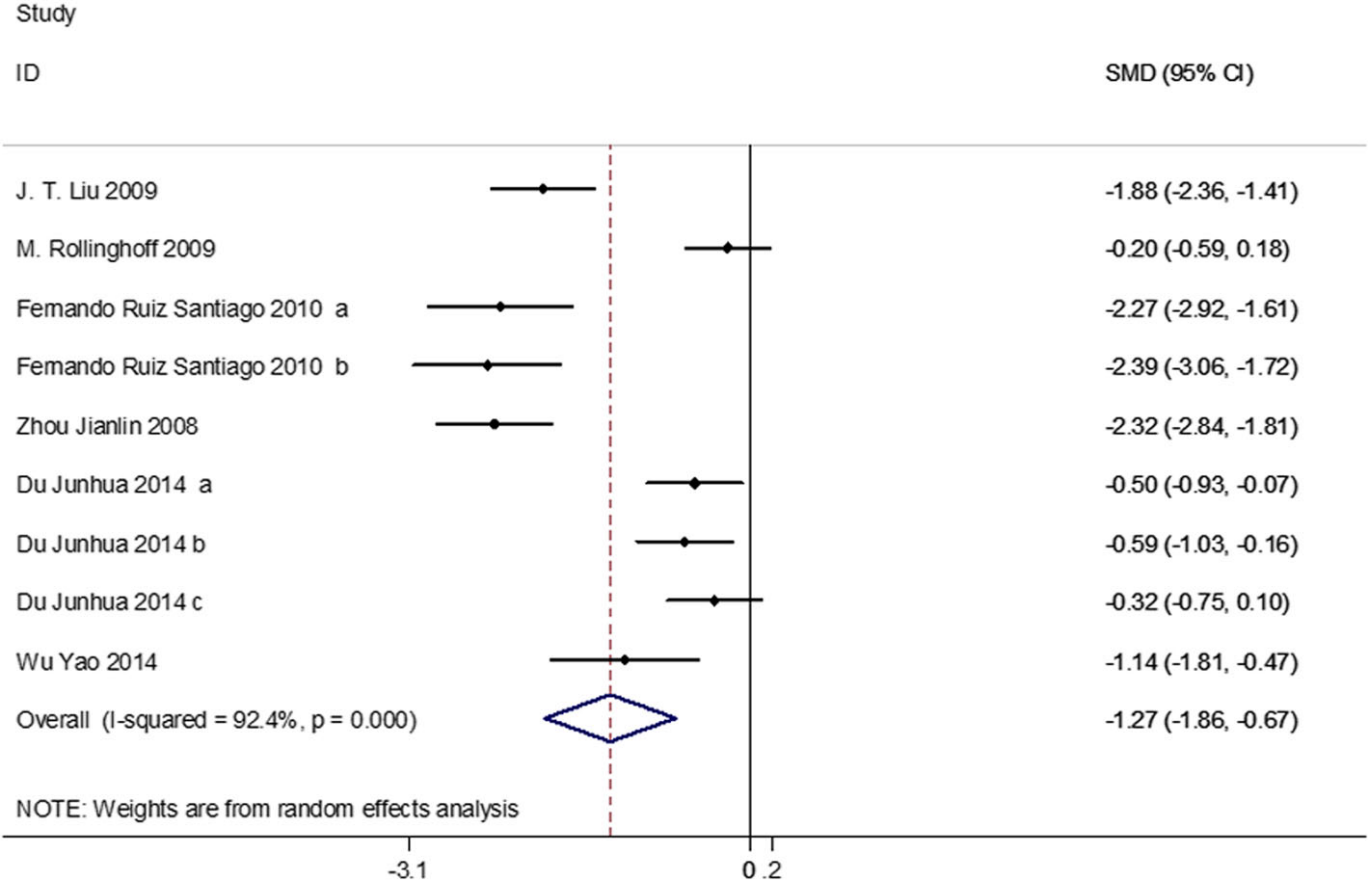
Forest plot showing the mean difference of vertebral body height between kyphoplasty and vertebroplasty

Six studies on 489 patients had preoperative and postoperative vertebral body heights. Based on the chi-squared test *P* value (*P* = 0.000) and *I*^2^ test value (*I*^2^ = 92.4%), we chose the random-effects model to analyze the MD of vertebral body height. The pooled results showed that compared with vertebroplasty, kyphoplasty significantly increased postoperative vertebral body height (SMD, − 1.27; 95% CI − 1.86 to − 0.67, Fig. 5).

### Cement leakage

**Fig. 6:**
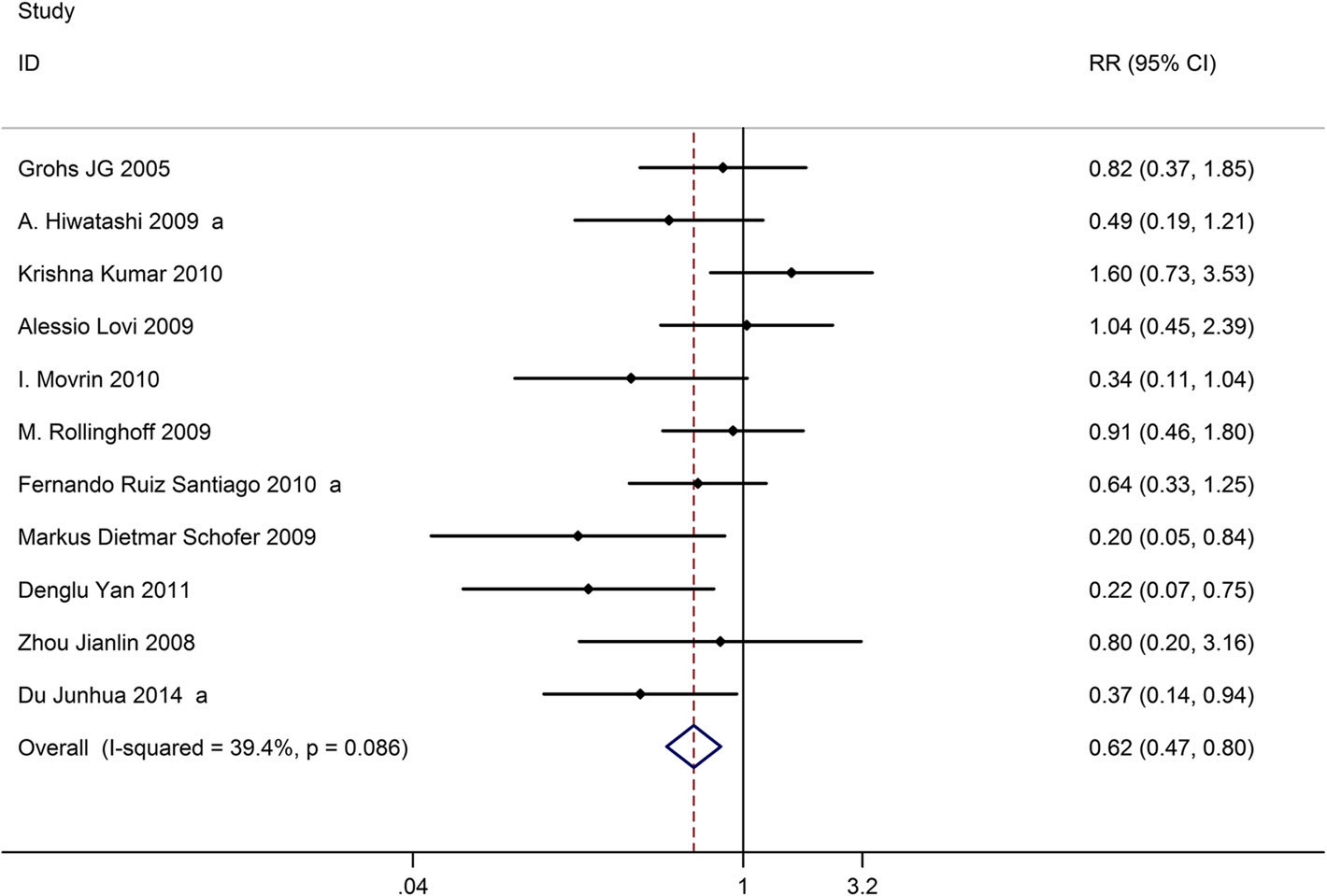
Forest plot showing the risk ration of cement leakage between kyphoplasty and vertebroplasty

Eleven studies on 1057 patients provided data on the incidence of cement leakage after the operation. Based on the chi-squared test *P* value (*P* = 0.088) and *I*^2^ test value (*I*^2^ = 39.4%), we chose the fixed-effects model to analyze the incidence of cement leakage. The pooled results showed that compared with vertebroplasty, kyphoplasty significantly decreased the risk of cement leakage (RR, 0.62; 95% CI 0.47–0.80, Fig. 6).

### Quality assessment and potential bias

**Fig. 7:**
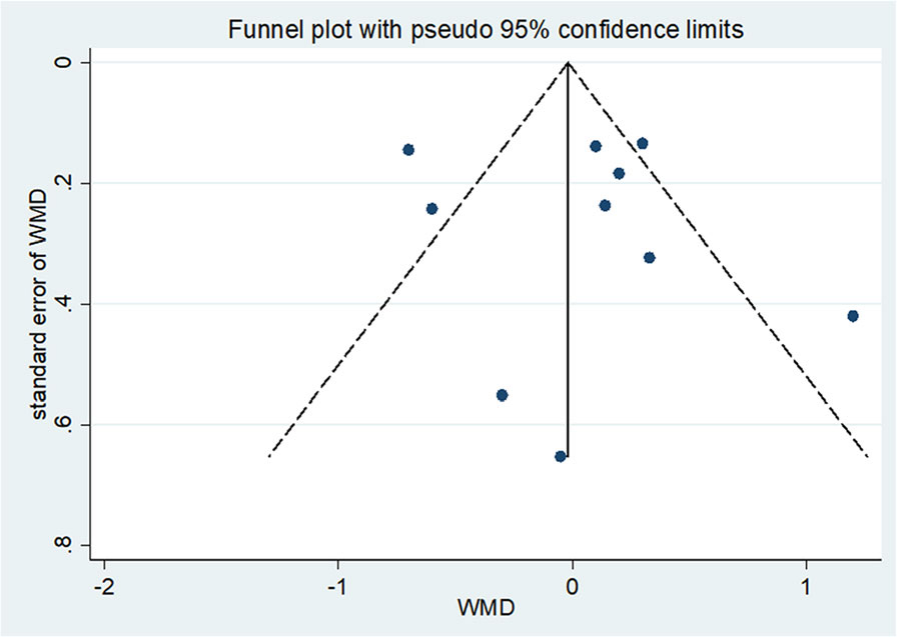
Funnel plot of studies included in the meta-analysis

Based on the inclusion and exclusion criteria, 16 articles were included in the meta-analysis. Quality and potential bias were assessed by funnel plot, Begg’s and Mazumdar’s rank test, and Egger’s test. The funnel plot for log WMD in VAS scores of the included studies was notably symmetrical, suggesting no significant publication bias (Fig. 7). In addition, significant symmetry was detected by Begg’s and Mazumdar’s rank test (*Z* = 0.36, *P* = 0.721). However, Egger’s test result showed no significant publication bias (*P* = 0.677).

## Discussion

Moderate evidence has been collected with funnel plot, Begg’s and Mazumdar’s rank test, and Egger’s test, showing no publication bias in the included studies; therefore, the results are credible. In this study, the outcomes include two categories, the radiographic difference and the clinical difference. The indexes of radiographic difference include kyphotic wedge angle, vertebral body height, and cement leakage. We found that compared with vertebroplasty, kyphoplasty could significantly increase postoperative vertebral body height and decrease the risk of cement leakage. The clinical outcomes include VAS scores and ODI scores. However, we did not find any significant difference in changes in VAS scores and ODI scores between the two groups.

Admittedly, there are several limitations in this analysis: (1) differences in the inclusion criteria and exclusion criteria for patients; (2) different patients with previous disease and treatments were unavailable; (3) all the included studies were English and Chinese publications being the source of bias; (4) the operating techniques in different studies were varied; (5) the low quality of included studies and the number of included studies is limited; (6) different types studies were included in the study; and (7) pooled data were used for analysis and individual patients’ data were unavailable, which limited more comprehensive analyses.

Several studies have been published in the past few years that performed similar meta-analysis of the efficacy of kyphoplasty versus vertebroplasty in the treatment of OVCF. Ma et al. [27] found that in the RCT subgroup, there were significant differences between the two procedures in short-term VAS, long-term kyphosis angles, operative times, and anterior vertebrae heights. In the cohort study subgroup, there were significant differences between the two procedures in short- and long-term VAS and ODI, cement leakage rates, short- and long-term kyphosis angles, operative times, and anterior vertebrae heights. Both kyphoplasty and vertebroplasty appeared to be safe and effective surgical procedures for the treatment of OVCF. Kyphoplasty tended to have more favorable outcomes than vertebroplasty for patients with large kyphosis angles, vertebral fissures, fractures in the posterior edge of the vertebral body, or significant height loss in the fractured vertebrae. Han et al. [1] reported that vertebroplasty is more effective in the short-term (no more than 7 days) pain relief. Kyphoplasty had a superior capability for intermediate-term (around 3 months) functional improvement. As for long-term pain relief and functional improvement, there was no significant difference between these two interventions. Consistently, both interventions were considered to have similar risks with subsequent fracture and cement leakage. Wang et al. [28] concluded that kyphoplasty and vertebroplasty are both safe and effective surgical procedures for the treatment of OVCF. Kyphoplasty has similar long-term pain relief, function outcomes (short-term ODI scores, short- and long-term SF-36 scores), and new adjacent VCFs in comparison to vertebroplasty. Kyphoplasty appears to be superior to vertebroplasty for the injected cement volume, the short-term pain relief, the improvement of short- and long-term kyphotic angle, and lower cement leakage rate. However, kyphoplasty needs longer operation time and higher material cost compared with vertebroplasty.

Longo et al. [29] reviewed conservative management of patients with VCFs, and found that no conclusions can be drawn on the superiority of cementoplasty techniques over conservative management. Denaro et al. [30] compared vertebroplasty with kyphoplasty in the treatment of VCFs and reminded us not to forget that for many years successful conservative management of vertebral fractures has been the standard of care. These conclusions are consistent with our findings that vertebroplasty and kyphoplasty had no significant difference in VAS scores, ODI scores, OVCF, and radiographic differences. Compared with the previous studies, some conclusions are consistent with Ma et al. [27] and Wang et al. [28]: kyphoplasty has some advantage in decreeing the kyphotic wedge angle, increasing the vertebral body height, and decreasing the risk of cement leakage than vertebroplasty. However, the conclusions about VAS scores and ODI scores were consistent with the studies of Vincenzo Denaro et al. [30].

## Conclusion

This systematic review and meta-analysis suggest that kyphoplasty confers benefits in decreasing the MD of kyphotic wedge angle and the risk of cement leakage, increasing the mean difference of vertebral body height. In addition, kyphoplasty has no statistical influence on the VAS scores and ODI scores compared with vertebroplasty. Therefore, kyphoplasty and vertebroplasty are equally effective in the clinical outcomes of OVCF, and radiographic differences do not make significant influence on the clinical results. Considering the limitations of this meta-analysis (different types of studies were included, and some studies were of low quality), more high-quality RCTs with larger sample size, multi-centric, and longer follow-up are warranted to confirm the current findings.

## Abbreviations

CI: Confidence interval
KP: Kyphoplasty
MD: Mean difference
OVCF: Osteoporotic vertebral compression fracture
RR: Relative risk
VP: Vertebroplasty
